# *One of the Many Things That I've Learned on This Journey…*: A Co‐operative, Narrative Inquiry With Two Care Partners of Spouses With Young Onset Dementia

**DOI:** 10.1111/hex.70827

**Published:** 2026-09-16

**Authors:** Elissa Burton, Vicki Barry, Bronte Parkin, Deborah Hersh

**Affiliations:** ^1^ Curtin School of Allied Health, Faculty of Health Sciences Curtin University Perth Western Australia; ^2^ Curtin enAble Institute, Faculty of Health Sciences Curtin University Perth Western Australia; ^3^ Brightwater Research Centre Brightwater Care Group Inglewood Western Australia; ^4^ Young Onset Dementia WA (YODWA) Impact Network Perth Western Australia; ^5^ Dementia Consumer Advisory Group Member, Curtin enAble Institute Curtin University Perth Western Australia

**Keywords:** Alzheimer's disease, care partner, posterior cortical atrophy, qualitative, Young‐onset dementia

## Abstract

**Introduction:**

Young‐onset dementia (YOD) is a term used when people demonstrate symptoms or are diagnosed with dementia before 65 years of age. YOD can be challenging to diagnose because health practitioners often do not initially consider dementia as the cause of someone's change in health or function in their 40 s, 50 s or even early 60 s. This can be the start of a complex journey for both the person living with YOD and their care partner. The aim of this study was to explore the journey for two care partners whose spouses were diagnosed with YOD.

**Methods:**

Co‐operative, narrative inquiry, which is a collaborative, participatory research approach was used. This enabled in‐depth reporting of the experiences of the two care partners, who were also research partners and authors on this project.

**Results:**

The story is told through a timeline lens where both care partners' narratives described their journeys prior to diagnosis, diagnosis, life after diagnosis through to transitioning to a care home. Both care partners experienced long periods of uncertainty as initial changes became evident, followed by frustration with health professionals leading to delays in onward referral and diagnosis. Both found the change of role to be confronting and their care for their spouse with YOD to significantly impact on their finances, work, social lives and wellbeing. Both worked hard to maintain their spouses' dignity and quality of life. They became innovative problem‐solvers and strong advocates. Both continue in advocacy roles at the local, state and national levels.

**Conclusion:**

Giving space to reflect on the stories of family care partners of people living with YOD is helpful in sensitising health professionals to the needs of their clients over time and highlighting the integral role of families in managing the condition.

**Patient or Public Contribution:**

Two lived‐experience contributors, both care partners of people living (or having lived) with young onset dementia, were involved as research partners throughout the project. They reviewed and edited the ethics application (including project proposal) to ensure the proposed research and participant materials were acceptable and understandable to care partners. They each participated in an individual interview (after providing written consent as required by the local Human Research Ethics Committee), shared their lived experience to help interpret and contextualise the findings, and provided detailed feedback on drafts of the manuscript. They also contributed additional information to strengthen the implications for practice and have been included as named authors on the paper in recognition of their substantial contribution and partnership in the project.

## 1 Introduction

Young‐onset dementia (YOD) is the term used when dementia is diagnosed for people under 65 years of age. According to Dementia Australia, around 10% of dementia diagnoses fall in this age bracket so, while rare compared to dementia in people over 65 years, the numbers are still significant. The global prevalence is around 119 people per 100,000 population aged 30‐64 years, which is approximately 3.9 million cases across the world [1], with around 29,000 in Australia [2, 3]. Types of YOD include Alzheimer's Disease, frontotemporal dementia, Lewy body dementia, alcohol‐related dementia, vascular dementia, posterior cortical atrophy, and dementia related to Huntington's disease and Parkinson's disease [4].

YOD can be difficult to diagnose and the time from symptom onset to diagnosis is often much longer than for older people with late onset dementia [5, 6]. People often require multiple consultations and testing from medical practitioners before a diagnosis is confirmed, which also increases the time to diagnosis [5, 6]. These issues appear to occur across the world, but are particularly prevalent in Australia as outlined by a research report on YOD in Australia [7]. Moreover post‐diagnosis supports and services are often lacking or not fit‐for‐purpose, catering instead to the life‐stages and needs of people with dementia aged ≥ 65 years [8, 9, 10]. Navigating the health and disability systems can be difficult. A systematic review by Mayrhofer and colleagues [11] found that age‐appropriate services for people living with YOD were variable in nature, lacked structure and were often only offered on a short‐term basis due to a lack of ongoing funds.

Studies exploring the carer role in YOD have been undertaken mainly in the United Kingdom, the Netherlands, United States, Canada, and Scandinavian countries [12, 13]. A recent scoping review found that care partners of people with YOD required education, social, emotional, psychological and practical supports including navigating services [14]. Qualitative research in Sweden has highlighted that care partners of people living with YOD often manage to care for them at home for longer than those caring for older people with dementia but also experience a reduced sense of wellbeing compared to those with later onset dementia [15]. Aspo et al. [15] interviewed 15 family members of people with YOD and reported their sense of responsibility to care well, but often also a sense of powerlessness and lack of control, particularly in seeking services.

There are relatively few Australian publications specifically in this area. Studies have included carers but have not specifically explored the carer journey with YOD [16, 17, 18, 19, 20]. Two questionnaire‐based studies identified that carers wanted improvements in early recognition and referral, access to specialist services and carer support [21], as well as help managing the psychological effect on the family, employment, finances, and service navigation [22]. A more recent Australian publication explored the role of assistance dogs in caring for people living with YOD. While beneficial, issues were raised about the implications for housing, the responsibility of keeping a dog in the family, and financial support from the National Disability Insurance Scheme (NDIS) [23]. Considering the particular disruptions facing families and carers when the person with YOD is of working age, when there may be parenting responsibilities, and when YOD is under‐recognised and relatively rare, a more thorough exploration of the experiences and needs of carers is required. Given health and social care service contexts differ across countries, even states or municipalities, there is value in understanding these experiences within a local Australian context. We have chosen to use co‐operative inquiry and a narrative methodology to detail the stories of two spouses of people living with YOD, who were cast into a caring role. As part of the co‐operative process of this work, we have adopted the term used by these spouses to describe themselves ‐ “care partners”. The purpose of our research collaboration is to move deeper and more personally into the issues highlighted in previous questionnaire‐based studies, and to prioritise the voices of care partners. This study enables close consideration of two of these stories of caring over time, offers a different way to understand the enormity of a YOD diagnosis and ultimately seeks to sensitise service providers to better support other care partners.

## 2 Methods

### 2.1 Study Design

This study used a co‐operative inquiry methodology [24], a collaborative, participatory research approach developed originally by Heron and Reason [25]. Their purpose was to extend different ways of knowing (experiential, presentational, propositional, and practical) and to make them intentional through the research process:

Everyone is engaged in the design and management of the inquiry; everyone gets into the experience and action that is being explored; everyone is involved in making sense and drawing conclusions; thus everyone involved can take initiative and exert influence on the process. This is not research on people or about people, but research with people.

[25, p.366]

This approach values lived experience, in this case, that of caring for someone living with YOD. Co‐operative inquiry includes participants as research partners from the start of a project, and through each aspect of data analysis, write‐up and dissemination. The research partners therefore sit on an equal footing with the academic partners and form part of the authorship team. Co‐operative inquiry sits comfortably alongside Narrative inquiry, a qualitative research methodology that enables understanding of human experiences through storytelling. As Kim [26] wrote: “Narrative inquiry, open‐ended, emergent and evolving, allows narrative inquirers to invite participants to become co‐researchers, co‐constructors, co‐narrators, and co‐storytellers” (p.99).

### 2.2 Recruitment

Two research partners with lived experience as care partners collaborated with two academics in this study. This decision was deliberate and reflected their public, proactive advocacy work, their desire to share their stories, and an opportunity to report rich narratives. This focus yielded different data to other possible qualitative research approaches, for example, interviewing larger numbers of care partners in order to find common experiential themes [27, 28]. We specifically chose to combine narrative inquiry with a collaborative cyclical process of action (the interviews) and reflection (the analysis), prioritising depth of engagement and shared meaning of the data over utilising larger samples [25]. This reflects a broader shift within co‐operative and participatory methodologies from research conducted *about* people toward research conducted *with* people, in which participants actively shape the direction and interpretation of the inquiry rather than serving solely as sources of data [24].

Both research partners live in Perth, Western Australia. Vicki's spouse, Michael, was diagnosed with YOD Alzheimer's 13 years ago and, at the time of writing, is living in residential care. Bronte was a care partner for his spouse, Glenda, also diagnosed with YOD, but with a rare type, posterior cortical atrophy (PCA). Glenda lived at home for 10 years and passed away after a further 3 years living in residential care. Both research partners already knew each other and the academic researchers due to their advocacy work for people living with YOD. The agreement for this approach was negotiated through personal contacts and a desire for all to be involved and to use this collaborative approach. Both Bronte and Vicki are authors on the paper and are Chief Investigators in this project along with the two academic researchers, Elissa and Deborah.

### 2.3 Ethics Considerations

Despite being co‐authors and investigators on this project, the research partners were both presented with a Participant Information Sheet and asked to sign a Consent Form prior to their interview being conducted. Co‐operative inquiry may not specifically require ethics approval [24] because of the nature of the relationship in this methodology. However, this traditional approach was favoured by the University ethics process particularly as the care partner authors, sharing their lived experience, would be fully identifiable to the readership. From this perspective, all identifying information of other people (beyond the couple) mentioned in interviews has been removed from accounts. All authors considered this carefully in writing up the study and the lived‐experience research partners were aware of their dual role in the project. Ethics approval was obtained from the Curtin University Human Research Ethics Committee (HREC) prior to commencing the study.

### 2.4 Data Collection and Analysis

An interview guide was initially developed by the two researchers, with the care partners providing their feedback on the initial draft. Questions were included if they were deemed important within the care partner journey. Both interviews were conversational and additional questions arose as stories unfolded. Interviews were run face‐to‐face for Bronte, recorded through a computer and audio recorder as a back‐up, and online via Teams for Vicki. One interview took 2 h and 15 min and the other took 2 h and 1 min. The use of computer while conducting the interview in person was to allow for automatic transcription of the interview. Each participant was interviewed separately, and the two researchers were involved in both interviews. In line with previous studies using co‐operative inquiry and narrative approaches, the interviews themselves were conversational in style and undertook a collaborative process of data immersion, interpretation and discussion. Transcripts were created from the online recordings and then recorded and written versions were compared to identify any transcription errors. The participants made several small editorial adjustments to their transcripts to clarify statements they felt were less than clear when originally spoken and to correct all factual errors or misinterpretations related to their statements.

Following the guidance of Riessman's [29] work on narrative methods, we used a narrative thematic analysis which focuses on *what* was told in the stories more than *how* those stories were told. While we looked for a thematic structure, it was important to hold each of the stories intact rather than focusing on the components. We deliberately chose extended quotes where appropriate to ensure experiences were shared in the person's words. We had a series of meetings to deepen the analysis and lead to a more interpretative account of the data, allowing the research partners to reflect on their stories as a shared narrative, woven into the paper and told by a *narrator*. Interpretive decisions were reached through dialogue and critical questioning with the participants, ensuring the narratives accurately represented their experiences. As noted earlier, the researchers and partners knew each other before commencement of the study in a context of strong mutual respect. The collaboration was a deliberate choice for all involved. From this perspective, what was included in the final draft, what was excluded, and how data were interpreted was a negotiated process. Different perspectives did arise, for example, around whether to include an incident that could have identified an agency. After discussion, the group finally agreed to omit this part of a story, despite its salience for the care partner. Other issues arose around balancing telling a complete story with word count restrictions. Through open discussion and collaborative writing and reviewing, these issues were resolved. Russ and colleagues^24, p.48^ argue that “participatory research powerfully counters… the inescapable biases of single authorship through an emphasis on participants' self‐representation in the research process and conclusions”. In this context, a formal process of member checking was not appropriate considering the collaborative nature of the analysis and write‐up. We continued to reflect, question and test the coherence and interpretation as it was written up collaboratively and reviewed through the writing process to finalise the narrative as a document that accurately captured the essence and experiences of the research partners.

## 3 Results

The result of the collaborative process was an interwoven, detailed account where both Bronte and Vicki independently presented their experiences through storytelling and a *journey* metaphor, as if travelling chronologically through time periods. This phenomenon of the way care partners talk about their journey of dementia has been reported before [30, 31]. The focus on preserving the integrity of the storytelling in this paper follows this journey but also reflects the key role of *illness narratives* in making sense of and managing life changes:

Disease happens in a life that already has a story, and this story goes on, changed by illness but also affecting how the illness story is formed…

[32, p.54]

Stories are important; they reflect what people choose to remember and to tell [33]. To capture the chronological flow of both interviews, we present the results as closely aligned to Bronte's and Vicki's accounts, and according to their broad categories of Pre‐diagnosis journey; Time of diagnosis and diagnosis reaction; Life after diagnosis; and Transition to Care Home.

### 3.1 Pre‐Diagnosis Journey

Both interviews included a detailed introduction to Glenda, Bronte's wife, and Michael, Vicki's husband, to set the scene for the unfolding narratives, and to contextualise the slow realisation that there was a change. Bronte said:

So, to begin with… my wife was working, a woman balancing a career, a family, a home. She was a high achiever. She had a lot of cognitive reserve. She was a school principal at a girls' private school at the time… She was highly regarded and well known in the education community…

(Bronte)

He described Glenda as being “run off her feet…” in her job after moving to this smaller school 2 years earlier from being principal of a much larger private school. Looking back, he wondered if her decision was a sign of her trying to cope with early cognitive changes: …Perhaps there was something going on, that she just couldn't get on top of the management of the new school…. However, the first insight that he had that something wasn't quite right with her came during the school's annual speech night, in a packed, large venue about a year after joining the school:

I was obviously in the audience with parents and teachers. And Glenda was on the stage giving a speech… at the lectern. And she was getting confused in reading the text and repeating it and shuffling around, having trouble with her mortarboard hat and academic gown slipping down. And she seemed to be quite restless. And I thought ‘what's wrong with you, why aren't you reading this thing properly?’ I felt sort of uncomfortable and embarrassed for her because it was so much out of character. Anyway, she got through it, and we talked about it afterwards and she said ‘I don't know, my hat kept on falling, I was distracted’. She didn't really know herself why she was unable to read the text… So that was the end of that year.

(Bronte)

Over the next 2 years, Bronte noted other incidents. Glenda misjudged a step on a stairway in the school's performing arts centre while at an art exhibition, fell and broke the bones in her feet; she struggled to manage routine, simple things, like putting food in the microwave oven, or cobbling together a recipe…; she seemed to hesitate when reaching for things; she drove her car into the wrong lane when turning right at a junction. Bronte said:

…towards the end of that second year, I did start to worry and 1 day at work, I had some spare time, and I went online and just Googled. I was typing up ‘difficulty with perception or perceptual ability’. I didn't find anything conclusive, and even if the word *dementia* had been mentioned, I would have completely disregarded it because I thought: ‘a woman who's 53; she's not going to get dementia, is she? That is an old person's disease.’

(Bronte)

For Vicki, who had spent 12 years raising her children as a single mother, her second marriage to Michael, who was working in local government as an experienced senior manager, “was going to be the next stage of my life… I was looking forward to this time”. She recounted:

Then 3 years down the track, I started to notice… little things. He'd forget his swipe for work. He'd leave glasses behind at the movies or the restaurant, and when he was cooking… he'd be continually asking me for certain ingredients that were in the pantry or the fridge, and I'd think ‘it's right there, you know, why can't you see that?’ So, after a while, I started to think ‘this just isn't normal… there's something really weird going on here’. I couldn't pin it. Like, I couldn't put it down to anything in particular. I began to think, oh, could it be a brain tumour? Like, you start to think all sorts of sinister things.

Never did I think dementia because, like everyone, we think dementia is reserved for the aged and frail, but the more I started to Google, try and explore, find what could possibly be going on here, more and more it pointed to dementia.

(Vicki)

Vicki went to see Michael's general practitioner to explain her concerns:

And he just said to me, ‘well, if Michael's got any problems, he'll discuss it with me’. He put his arm around me and guided me out of his office and just said ‘go home and be a good wife’. I was speechless. Seriously, I had no words. I was flabbergasted. So, I went home feeling powerless. I thought I was being a good wife actually’.

(Vicki)

Vicki described this period as feeling very alone and “looking for reassurance”. She raised her worries with Michael who she believed was aware of these signs but was hiding them. She tried to speak to colleagues and old friends of Michael's who had known him for a long time, even to the pastor at the local church “as a trusted person that I could confide in, he even put his hands up and said ‘I don't want a bar of that’”.

And I think that's the whole thing because it's scary. It's fear. And you know, what if we've got this wrong? Like you've got no definitive diagnosis, like, it's a hunch, there's nothing to go with. So, I really found myself out on a limb where I had no one.

(Vicki)

So, she contacted Alzheimer's WA (then the peak body) and “was very fortunate to meet a lovely woman who allowed me to just let it out… she recognised the grief”. This session affirmed her concerns, gave her permission to consider whether to stay in the relationship considering she knew “what was down the line”. Vicki “decided to go to Michael with some non‐negotiables”. He would need to go to his doctor, get a formal diagnosis and then go to counselling to “redefine our relationship and the way forward”. By this time, Michael was having difficulty managing his tasks at the computer and was clearly struggling to cope at work.

He ended up coming home 1 day and said ‘oh, I'm on a contract for a year’. And in that process, he had relinquished permanent employment and was on a 1‐year contract… And he had 884 hours of sick leave accrued. Guess what, all that went out the window and a year later when he thought he was being recontracted, he went to a surprise leaving party [his own]. He came home absolutely gutted.

(Vicki)

Michael was 62 when he lost his job. He had not understood the implications of the move from continuing employment to the new contract.

### 3.2 Time of Diagnosis and Diagnosis Reaction

For both Bronte and Vicki, the slow growth of concern, of uncertainty, and search for answers, eventually reached a turning point which thrust them into seeking a diagnosis. This turning point related to an incident or event prompted by others who were also starting to notice something unusual:

When you get to the diagnostic journey, you kind of look back and you think, well, how come we didn't know that or how come we didn't see that? Or we did actually, but we didn't think much of it. Oh… it's just one of those days, you know, having an off day or, you know, it's just a part of life. It doesn't sort of add up, until it gets to a point where maybe others start to observe changes…

(Bronte)

Over and above the previous increasing worry, the couples were tipped over from one phase of the journey to another. For Vicki and Michael, this was the loss of employment and the feeling of rejection from friends and colleagues. For Bronte and Glenda, the tipping point was a holiday that Glenda had with her sisters. Bronte explained:

… one of her sisters, a former nurse, rang me 1 day and she asked ‘have you ever noticed something strange about Glenda? You know, like something odd?’ And I said, ‘you know, I think I know what you're going to say’. And she confirmed similar observations. And she said ‘well, we were in the kitchen, and we were getting meals ready. And we said to Glenda, you put this in the microwave. And Glenda was standing in the middle of the kitchen… looking around, thinking, you know, where the hell's the microwave? What is the microwave? What does it look like?’ So, I then told her what I knew and her sister said ‘she might have a small tumour on her brain affecting her eyesight or whatever. Just go off to the GP and talk about it.’ … So, her sister convinced her to go to see a GP…

(Bronte)

Visiting the GP was challenging. Bronte said: “we were a bit upset when we walked out because the female GP was a bit dismissive and even suggested Glenda was a ‘spoilt’ woman who had most things done for her given her role as a principal and with a house‐husband at home”; although she referred Glenda for an MRI and onto a neurologist. This did not show anything so at least they knew it was not a tumour. They then waited another couple of months to see the neurologist who “listened intently”:

He was intrigued by my observation that this was a perceptual issue. So the thing that I learnt then was how important it is for the person closest to the person ultimately diagnosed with dementia to be able to tell the story about what's changed, what's changed in the behaviour, what have they noticed that's different from what used to be before because that's really a major clue to helping the clinicians do the diagnosis. And I guess that's probably something that can sometimes be difficult for the person themselves, if they even mention the word dementia that they feel ashamed and embarrassed about having that discussion. So, it can be an uncomfortable and awkward thing for some people to handle it. But I do think that those closest to the individuals should really be there and the doctors should actually be asking them more questions than the person themselves.

(Bronte)

Bronte played a key role in the consultation, directing the neurologist to the issues of concern. He asked the neurologist to use a painting of people walking along a path in a park, that hung on the wall of his consulting room as a test for Glenda:

And I said ‘if you ask Glenda just to point to the people in that picture, you know, do that now’. And so he said, ‘Glenda, can you point to any people in the picture?’ And she couldn't discern the figures from the surrounding landscape in the picture… it was a bit like the old saying goes, ‘she can't see the wood for the trees.’

(Bronte)

Bronte noted that the neurologist then ordered new brain scans for Glenda and asked a clinical psychologist in the practice to develop a special version of a diagnostic test that could cater for Glenda's high level of cognitive functioning. Bronte said:

Meanwhile, I had been, in my usual way, Googling around, trying to find out things about perception and dementia. And I came across ‘posterior cortical atrophy’. And it had a list of symptoms. I printed this off and 1 day I gave it to Glenda and I said ‘here read this’ and she was mortified seeing these examples of what cognitive and behaviour changes were occurring or could occur, so I said ‘I think that's what you are going to be told next week when we go and see the specialist’…. and that's what happened.

(Bronte)

The time from the initial visit to the neurologist to the delivery of a diagnosis was 6 months. Glenda was still working during this period. She had earlier confided in one of her close colleagues that she was undergoing some tests, and that, as she did not want to make any errors that might undermine the school, her colleague should alert her to any issues that arose. Her colleague later admitted that the staff were “starting to talk” and doubt Glenda's capability to lead the school. At this point, Glenda contacted the chairperson of the school board who suggested she take sick leave. Bronte's account of this was that the chairperson said: “They'll probably think you've got breast cancer or something” and “made light of it” – a revealing comment where breast cancer is “light” and less stigmatised compared to a diagnosis of dementia. During this period, Glenda also made the decision to stop driving. When the couple finally received the diagnosis of posterior cortical atrophy from the neurologist, he suggested they label it as a “functional blindness”. Bronte recalled that the neurologist said: “Pack your bags and start travelling because I can't guarantee how much you're going to be able to see or recognise within a year or two or whatever”. The couple were not offered any brochures or information about where to get support:

He said ‘goodbye and we'll see you in another month’… we drove home in silence, then we got our two teenage sons and sat them down to tell them. They knew of course… I'm getting a little bit emotional about it because I'm just thinking now of the reaction of our eldest son… he got really angry… the younger son, he was quiet. He didn't really say a lot. He just took it in.

(Bronte)

Vicki's 5‐year journey to a diagnosis for Michael was complicated by a series of unnecessary medical assessments attributed to a practitioner who was later found to have engaged in misconduct in other aspects of their work. She lodged a grievance. She and Michael attended a meeting to explain their concerns, and it was then that the practitioner announced the diagnosis:

And I turned to the Geriatrician, I said, ‘I'm sure in all your 30 years of experience that, on the first occasion you saw Michael, you would have known, without doing any assessments, what was going on for Michael’. And [the practitioner] said ‘Oh yes, he's either got X, in which case he's got 5 years to 7 years to live, or he's got Y and he's probably got 7 to 12 years’. And Michael… sitting there [until that point] thinking there's nothing wrong with himself.

(Vicki)

The timing of the diagnosis for Michael was difficult in terms of structural changes with the introduction of the National Disability Insurance Scheme (NDIS) in Western Australia. Vicki said: “We were all floundering at that point. And even service providers really didn't know what the landscape was going to look like”. Michael missed out on a NDIS funding package by 3 months:

We got an ACAT [Aged Care Assessment Team] assessment and had to wait another 2 years for a Level 3 package to become available… I was only working 20 h a week by that stage because Michael needed support and help, and eventually I had to give up work. So, the impacts were huge for us.

(Vicki)

Despite the turmoil of this period, Vicki found the diagnosis useful as it finally gave her a position from which to move ahead, including through accessing counselling services:

At least we had something in black and white… So, the denial for Michael was no longer. Like we have something real going on here… If you don't know what you're dealing with, you can't manage it, right? So, for the first time, I had something tangible, could manage, and that's what gave me, you know, to say to Michael ‘it's just not negotiable anymore. This is how we have to go forward’. I was mindful if it was going to work, we're in partnership together. So, the counselling was really important. We had to be on the same page, like that's the only way we would get through it.

(Vicki)

### 3.3 Life after Diagnosis

Vicki felt isolated and ploughed her energies into establishing a network with other families affected by young onset dementia. She noted how she drew on her own strengths at the time:

I don't know where you go… when you are relying on others. I'm just glad that I've got that wherewithal. And because of my history and issues I've had to work through in my own life, I've acquired the resilience and ability to hold space for myself, allow time and not fall apart. Because I tell you what, it's not for the faint hearted.

(Vicki)

The network, partly a response to Vicki's concerns that family members were not always valued by health professionals (“we just get overlooked…”), became a deeply valuable source of support for Vicki:

We started to meet on our own. And it's interesting out of the 30 founding members… Michael is one of three still alive; the rest have all died. But we still stick together and meet regularly for a catch up. We've got a group going now called the *YODows*. They're all the widowed care partners… But we've just formed that bond because it's something, it's almost unbreakable. Because unless you're going through it yourself, no one gets it. Seriously. No one gets it. The YOD Peer and Family Support Group now has 160 members.

(Vicki)

Vicki was upfront and organised. She made sure she attended regular counselling sessions. She organised documentation of wills and advance care planning, verifying with Michael what he wanted for his own end of life care. She said:

I had to sit my kids down because I said if anything happens to me, this is what happens for Michael. So, they're substitute decision makers. We've had to go through all of that… I think as soon as you can is a good time to do it because you just don't know what tomorrow will bring… dementia teaches us that.

(Vicki)

Despite her energetic desire to develop new social network opportunities where previous ones had faded away since the diagnosis, and to maintain Michael's quality of life, Vicki also attended to self‐care where she could:

One of the many things that I've learned on this journey, which has made me a much better person, is the willingness to let go of control. I was one of those who liked to organise, to be in control. But the one thing about living with dementia is it's so unpredictable. No two people are alike… there's so many variables. You'll never have control … And that's why I encourage the care partners to take it easy. Take some pressure off yourself. Learn to live in the moment and take things as they come.

(Vicki)

Bronte cared for Glenda at home for 10 years following her diagnosis and resignation from her school. In the first year, Bronte was able to continue working and Glenda could still go out unaided in the local community. The plan was to continue working until their youngest son had finished his schooling. Bronte then was granted carer's leave for the next 2 years and finally took early retirement. Glenda retained clear insight into her own condition for many years, which meant she was often frustrated and angry about her situation. However, she had previously been a social person with a positive outlook, and Bronte felt these traits continued despite her dementia. He reported managing her frustrations by working to keep her stimulated and busy, reading the newspaper to her each day, explaining things they watched together on television or at the cinema. The couple were often active, travelling, and engaged in social activities. Bronte organised music sessions, something that Glenda enjoyed, and these became particularly important as her condition deteriorated. As a previously prominent person in the education community, Glenda was approached by a local newspaper for an interview a short time after leaving her position as a school principal. This publicity led to an invitation from Alzheimer's WA, a key dementia support organisation in Western Australia at the time, for the couple to take an advocacy role with them. Glenda then became the WA representative for the inaugural Dementia Advisory Committee for Alzheimer's Australia (now Dementia Australia, see supplementary document for advocacy work). Bronte recalled:

She was very upfront about her diagnosis. She said so in one of the Alzheimer's WA videos, she said ‘I tell people I've got dementia. You know, I'm not afraid of it. I have a very good life now… I want to enjoy the rest of my life.’ …She had a very positive outlook for much of the time.

(Bronte)

Bronte described himself as a “fairly organised kind of person… a lot of planning and preparation before I do anything”. He organised their wills and enduring powers of attorney. He responded to needs as they arose. He explained that visual processing, for a while, was the main issue that Glenda was facing although she became less able to manage writing, spelling, calculating numbers, and reading as time went on. When they went out, she would greet people who knew her through the school community and chat with them: “…then we'd walk on a bit and I'd ask, ‘who were they?’ and she'd say ‘I have not got the faintest idea’… She had lost that visual facial recognition”. Eventually, Bronte sought help by contacting a support organisation for people with visual impairment, and they provided a bright yellow badge for her which stated, “Visually Impaired”. He found this helpful in many situations. For example, in airports, when walking through security, he was told he could not accompany Glenda through scanning machines, because even a short separation caused her to become agitated. However, he explained that when presenting her as a person who was blind rather than as someone with dementia, “everything opened up” ‐ the couple were offered help and were allowed to stay together through airport screenings. Bronte joined the Qantas club to give better access to a disabled toilet without the queues and to try to avoid Glenda needing the toilet on the flight itself. Toileting was a particularly salient issue, for example, when out in the community:

The difficulty with “disabled toilets” is a big thing for a male care partner taking someone with dementia to a female toilet. If there's no “disabled toilet”, it's quite challenging, because you have to explain because you get a look that says, ‘What the hell are you doing here? Get out of here’ kind of thing. And I say ‘my wife has dementia and she needs assistance’. So I've done that, you know, and I've been discreet about wherever I put my eyes…. Also at “disabled toilets”, we did get …frowned upon with looks that query, ‘Why are you two walking in?’ Because Glenda didn't look as if she had dementia and yes, most people who have dementia don't look as if they've got dementia… there's those sorts of issues you deal with… if they sort of look so‐called *normal*…

(Bronte)

Bronte took on all the household chores “like washing, cleaning, shopping, etc. Doing all that didn't phase me”. It was also important that he maintained Glenda's dignity:

I took responsibility for Glenda's grooming and dressing… I said to myself, I'm not going to let my wife become an undignified person. She was quite an attractive woman… she was like a striking person because of her height… she could shower herself and toilet herself pretty much right until the end… but she could not choose her clothing, or get herself dressed and looking smart… I would choose everything right down to the matching earrings and, you know, appropriately for the occasion. I did this because I had a stereotypical image of some old lady with dementia slopping around in a tracksuit and slippers. That's not a true image I suspect, but I didn't want Glenda to degenerate into that kind of an image.

(Bronte)

He also spoke about the challenges of clothes shopping for Glenda and how he gained the confidence to explain why he needed to assist:

And the thing is, some of those shop assistants were remarkable. They'd say ‘that's OK, we'll take her’ and ‘my grandmother's got dementia, or my sister had dementia.’ So all of a sudden, you realise there are people, once you reveal the condition, that are there to help. There were a few times when, buying clothing for Glenda, I would be rifling through the racks with her and saying, ‘oh, here, so, you know, size 14 or 16… do you want this red one, blue one, pink one?’, whatever. And some women, other customers, would eye us off with a look that said, ‘this is a very controlling husband’, you know, dictating terms to his wife. But I never said anything to them. I just carried on with the shop assistants when she went in to try the clothes on behind the curtain or the door… Initially I was a bit coy about saying ‘dementia’. I would say ‘my wife has an issue with colour recognition, which is why I've chosen things.’ Then I got over that, and I would say, you know, she actually has dementia.

(Bronte)

Managing life with a spouse with dementia was characterised by lack of stability, the need for strategies and carefully planned routines to shift and accommodate to the inevitable decline of function. Examples of this incremental shift could be seen in various situations: Glenda initially walked independently to her local shopping centre “until she got to the point where she couldn't even get back home”. Bronte then walked with her when she went out; Glenda joined a community choir, and the organiser printed her music in a large format. When this became too difficult, she joined a special dementia choir. Bronte described other accommodations he made over time so that he could go for 2‐h periods to his gym. Initially, as Glenda enjoyed listening to music and would wait home alone for him. For a short time, she would also occupy herself with books with enlarged font on an iPad that he had arranged. When that became too difficult, she moved on to listening to talking books until operating the player became difficult, and then finally only listening to the radio or continuous Youtube music on the TV. As the dementia progressed, Bronte ensured that all gates and doors to the outside were locked when he went out. Finally, in her last year at home, he had to arrange for a paid carer because he “couldn't trust leaving her alone at home”.

Bronte explained that one of the hardest issues as a care partner was managing disturbed sleep. The “constancy of being woken at night” had left him with an ongoing problem of sleeplessness:

Towards the latter part of that 10‐year period, Glenda was waking up at night. She wasn't necessarily going to the toilet; just waking up and getting out of the bed and wandering around because this happens with people with dementia… a terrible term called *sundowning* where the circadian rhythms get out of whack, and they might think it's daytime when its actually night‐time. So, if you're sharing a bed, and someone is moving around a lot… you're on edge all the time, asking yourself ‘Is she going to get out of bed? What does she want?’ You're ready to take action. Yeah. So that's probably one of the major downsides of being a care partner.

(Bronte)

### 3.4 Transition to Care Home

About a year before Michael moved to residential care, Vicki was monitoring her ability to cope. She said:

I made a promise to myself that… if I ever felt that I was going to get resentful or to a point where I just couldn't do it anymore, I would start to look at options then. Because I just knew if I wasn't good in myself, I couldn't be a good care partner as well.

(Vicki)

After much searching for options, she started Michael with a 2‐week period of respite, then sometime later, a 5‐week break. She organised for him to have regular visitors during that time from old friends and his support worker. During a third period in respite, Michael's mother died. Vicki had felt uncomfortable about the prospect of transitioning Michael permanently into care while his mother was alive, but the timing now felt appropriate. She said she did not wait for a “crisis point”. She discussed it with Michael:

He graciously tried to make it easy for me and agreed. He knew I was at that point. So, I think he still had that capacity himself to realise that it was the best thing for both of us.

(Vicki)

However, the transition to residential care brought new stresses. Michael was placed in a large residential facility where he had previously had a period of respite. It was not long before Vicki started to observe practices which she found “quite distressing” such as seeing staff walk past a screaming resident who had slipped out of his wheelchair or finding faecal matter on chairs. She described a lack of consistent staff, little personalised care or one‐on‐one interactions. She saw few visitors for other residents. Michael was often under‐dressed in cold weather, overly hot, or dressed in other people's clothes. She said:

And honestly, after a year, they still couldn't give Michael a black cup of coffee with no sugar. I mean, how hard could that be? Seriously, I'd go there and it was always milky tea with sugar. Everyone gets the same thing.

(Vicki)

The facility then had an outbreak of scabies: “they're all scratching. And Michael, florid. And I'm saying to them ‘you've got a contagion. What are you doing?’ And they didn't respond to that for ages because they really didn't know what to do”. Michael also contracted folliculitis. Vicki was losing sleep and searching for an alternative. Through a contact, she discovered a vacancy in a small group home established for people with dementia, co‐located in a retirement village closer to home. While not entirely without issues, she was intensely relieved by the change:

The residents are stimulated, engaged in healthy, meaningful ways. You know, it's very quiet. Any challenging behaviours? I don't see any behaviours. In fact, they're all very… calm. Honestly, it's just lovely.

(Vicki)

Characterising this period, Vicki spoke of the balance between recognising staff who were trying their best in difficult circumstances and calling out poor management. She was also aware of Michael being relatively young among other residents who were mostly older, and her responsibility to protect him within the system: “You are the advocate. You are the voice. You are absolutely looking out for them”.

Towards her last 2 years at home, Glenda's behaviours started to change more dramatically. Bronte reported: “and then in the beginning of that 11th year she went downhill really quickly”, and she was prescribed two psychotropic medications. He later learned that one of these created a movement disorder, known as dyskinesia, and had a falls risk side effect. Bronte noted:

And she had a couple of near misses with minor stumbles outside… and then she had a serious fall inside on the timber floor in the family room one evening when I was busy getting dinner ready. She was sitting watching television but had been very agitated that day… And then, all of a sudden, I heard a scream and found she'd fallen onto the floor… She was taken off by ambulance to hospital. She'd fractured her pubis rami bones. So that was the end of it. And she never walked again.

(Bronte)

After 5 weeks, Glenda went from the hospital direct to residential care. This was not an easy decision, but with her immobility and dementia, Bronte needed the support:

I had to think long and hard about that decision. Our family on both sides said, ‘look, you know, you've done 10 years of caring, it's time for you to now hand over to professionals’. So, I guess it was probably about the right time because… I suppose I was getting weary. You know, not physically exhausted, but mentally exhausted.

(Bronte)

By this time, the couple's adult children and their partners had moved interstate for their careers. Visits were difficult over the period of the COVID pandemic because borders between states were closed for some time. However, the family had many FaceTime calls and although Glenda could not “see” her children, they could see her. Initially, she spent a lot of her time in bed because of the dyskinesia and fear that she might slip from a nursing chair until Bronte finally convinced the care home to take the risk so she could be taken outside around the home's courtyards and verandahs. Throughout this time, she continued to enjoy listening to music.

We used to say ‘well she's got no quality of life in there. She is in a nursing chair for most of the day other than being in bed, she can't see anything or anyone, and she's in her room. She didn't have any socialisation with the other residents. She couldn't see them and they were probably not interested in her anyway. So, she had a fairly insular existence other than what she could hear on her Youtube music channels and when she had visitors like me or friends and family.

(Bronte)

Glenda spent her final almost 3 years in residential care. The transition from home to care was a deeply significant point for Bronte:

So… it really hits you. You realise that your life partner is gone. I mean, it's not the same as I imagined death would be, but you do go back to an empty house. So, when she finally died and people were being empathetic and sympathetic, saying ‘You must be lonely’, I responded by saying ‘yeah, but you know, it happened 3 years ago when she first left home’, you know? That's when it hit, hit me most, I suppose, that absence of someone being at home when you come home.

(Bronte)

## 4 Discussion

This study has followed the stories of two care partners of people living with YOD, giving detailed insight over time to their experiences of caring, of coping and of loss. While narratives of caring for people with dementia are not uncommon [34], these are less so in the context of YOD [27, 35], particularly in the Australian context, and few studies have taken the methodological approach used here combining co‐operative and narrative inquiry to explore this experience. These narratives raise some similar issues to those reported in previous qualitative studies such as balancing care of children with care of the partner, the need to be an advocate, social isolation and the desire to connect with others going through similar challenges, and physical and emotional exhaustion [15].

However, important insights have also surfaced through this work. First, spouses or partners are not expecting cognitive changes in someone so young, someone who is working. The period of time between noticing a change and seeking a diagnosis is long and unsettling and this was clear for both Vicki and Bronte. Their accounts highlight a slow realisation that perhaps the signs of cognitive changes are significant but in both accounts in this study, it was the reactions and concerns of other people, perhaps other family members or work colleagues, that prompted action. The impact of YOD was profound for many reasons: the financial implications because everyone was initially working then they all weren't [36]; managing the community reactions of shock, disbelief, and stigma in a society that assumes dementia is only a problem for the retired or older people; managing the person's reactions about their own changing level of functioning – including denial and grief [37]; coping with the impact on teenage children and young people at the time of diagnosis and the anger, distress and loss for them [38].

Second, this work highlights the important role of the spouse or partner as a co‐diagnostician. Specialist clinicians may view spouses as family members who attend sessions to receive education and support for the patient and for themselves in coping with the caring role. However, these stories demonstrate the value family members bring to assessment sessions as the experts in the functional changes and behavioural signs that might lead to a speedier and more accurate diagnosis. The initial dismissals that both Vicki and Bronte experienced from their General Practitioners were unhelpful and upsetting. The combination of waiting, or even reluctance, to discuss growing concerns about possible cognitive changes in a spouse with subsequent dismissive reactions from a General Practitioner contribute to emotional distress as well as diagnostic delay. We suggest that improved education is required for frontline healthcare workers such as General Practitioners so that they can recognise the growing concerns of family members, incorporate them into their diagnostic and referral processes, and give them legitimacy. Greater recognition is required that YOD is rare and particularly disruptive with the phase of working life with profound long‐term impacts on younger spouses, families, finances and future planning. The stories in this study show that clinical encounters were more productive when clinicians listened to accounts of behavioural and cognitive change and took them seriously. Early involvement of other members of the multidisciplinary dementia team would also be useful, for example social work in managing concerns related to changes in work status, finances, and future planning. This work highlights the role of the family member as careful observer, and as a resource and a partner for clinicians. These findings are similar to carers' experiences in other countries [15, 39]. Third, this study shows the importance of problem solving, even prior to diagnosis. Both Vicki and Bronte spoke of these many times across their journeys. Problem solving in daily life situations was particularly important given the lack of education, awareness, and understanding of YOD by health practitioners and the community generally. Previous research suggests that being resourceful is a necessity for this group considering the lack of tailored supports [27]. Post‐diagnostic services are limited and at times can be difficult to find. This is not specific to the Australian context; it appears to be an issue for people living with young onset dementia and their care partners across the world [15, 39]. However, being resilient and finding different ways to achieve an outcome became an important part of the journeys described here.

Fourth, we have highlighted the profound experience of transitioning a loved one, still relatively young, into institutional care when those services are stretched and often under‐staffed or under‐funded. Over the last decade in Australia with the changes in NDIS, it appears carer experiences may be changing with the level of engagement in peer support reducing [40]. Funding now enables some care partners to continue working. Having multiple care workers involved in funded care plans can be experienced as difficult for the person living with young onset dementia and may lead to transitioning into residential care earlier [40]. These experiences, and those leading up to them, pivoted Bronte and Vicki into active advocacy roles, speaking up for those who were becoming increasingly vulnerable, and becoming a source of support for others in similar situations. The strength of bond and friendship with the YOD Peer and Family Support Group was clear in this story, and both Bronte and Vicki continue to advocate and raise awareness at local and national levels.

Finally, this work personalises the experience of family members of people with YOD and allows the reader to follow what is often a long and difficult journey. While there is far more to these stories than can be reported in a single article, this approach complements and strengthens the findings of other studies of the needs of family members of people with YOD and gives those with lived experience the opportunity to be heard on their own terms and in their own words.

### 4.1 Strengths and Limitations

Both lived‐experience partners are articulate and highly educated people, and their experiences with YOD have thrust them into prominent positions as advocates within the dementia community. Sharing their stories adds considerably to this study but we recognise that their experiences may differ from those of carers with lower health literacy, fewer resources or less access to advocacy networks. This work does not suggest all experiences will be the same. Focusing only on two people with lived experience could be viewed as a limitation. However, the two care partners in this paper have listened to and recognised the value of stories from the many people they have met and therefore see themselves as a “voice for others” on this journey. In addition, our study design fits with co‐operative inquiry and narrative methodologies and sharing personal stories in the detail afforded here may resonate with readers and sensitise those who work with them.

## 5 Conclusion

This study resulted in detailed accounts of the journey over time for carer partners of people living with YOD, and an opportunity to explore the impact on families of the condition. This research seeks to move beyond simply positioning care partners and families as receivers of information and support by expert clinicians. Instead, they may act as supports for diagnosis and for decision‐making through the journey of care. The narratives in this co‐operative inquiry provide an opportunity to highlight gaps in services within an Australian context, unmet needs, and the strengths that care partners and families can bring as they travel alongside their loved one with YOD.

## Author Contributions

**Elissa Burton:** conceptualisation, funding acquisition, data curation, writing – original draft, writing – review and editing, methodology, formal analysis, investigation, project administration. **Vicki Barry:** writing – review and editing, investigation, project administration. **Bronte Parkin:** writing – review and editing, investigation, project administration. **Deborah Hersh:** conceptualisation, writing – original draft, writing – review and editing, methodology, formal analysis, data curation, investigation, project administration.

## Ethics Statement

Ethics was approved by the Curtin University Human Research Ethics Committee [HRE2024‐0585]. Written consent to participate in this research project occurred prior to any data collection and has been included within the manuscript.

## Conflicts of Interest

Although V.B. and B.P. are both participants and authors on the paper their roles have been instrumental in shaping this work and we do not view this as a conflict of interest given the transparency provided across the paper.

## Data Availability

The data that support the findings of this study are available from the corresponding author upon reasonable request.
